# A phase II study of the histone deacetylase inhibitor vorinostat combined with tamoxifen for the treatment of patients with hormone therapy-resistant breast cancer

**DOI:** 10.1038/bjc.2011.156

**Published:** 2011-05-10

**Authors:** P N Munster, K T Thurn, S Thomas, P Raha, M Lacevic, A Miller, M Melisko, R Ismail-Khan, H Rugo, M Moasser, S E Minton

**Affiliations:** 1 Division of Hematology and Oncology, University of California, San Francisco, 1600 Divisadero, Rm A719 Box 1711, San Francisco, CA 94143, USA; 2 H. Lee Moffitt Cancer Center and Research Institute, 12902 Magnolia Drive Tampa, FL 33612, USA

**Keywords:** histone deacetylase, HDAC, HDAC inhibitors, breast cancer, oestrogen receptor, anti-oestrogen therapy

## Abstract

**Background:**

Histone deacetylases (HDACs) are crucial components of the oestrogen receptor (ER) transcriptional complex. Preclinically, HDAC inhibitors can reverse tamoxifen/aromatase inhibitor resistance in hormone receptor-positive breast cancer. This concept was examined in a phase II combination trial with correlative end points.

**Methods:**

Patients with ER-positive metastatic breast cancer progressing on endocrine therapy were treated with 400 mg of vorinostat daily for 3 of 4 weeks and 20 mg tamoxifen daily, continuously. Histone acetylation and HDAC2 expression in peripheral blood mononuclear cells were also evaluated.

**Results:**

In all, 43 patients (median age 56 years (31–71)) were treated, 25 (58%) received prior adjuvant tamoxifen, 29 (67%) failed one prior chemotherapy regimen, 42 (98%) progressed after one, and 23 (54%) after two aromatase inhibitors. The objective response rate by Response Evaluation Criteria in Solid Tumours criteria was 19% and the clinical benefit rate (response or stable disease >24 weeks) was 40%. The median response duration was 10.3 months (confidence interval: 8.1–12.4). Histone hyperacetylation and higher baseline HDAC2 levels correlated with response.

**Conclusion:**

The combination of vorinostat and tamoxifen is well tolerated and exhibits encouraging activity in reversing hormone resistance. Correlative studies suggest that HDAC2 expression is a predictive marker and histone hyperacetylation is a useful pharmacodynamic marker for the efficacy of this combination.

Despite a decrease in incidence, an expected 194 280 women will present with breast cancer in the United States in 2010, resulting in over 40 000 deaths (Jemal *et al*, 2010). In more than two thirds of these women, tumours express either oestrogen receptors (ERs) or progesterone receptors (PgRs), which are frequently less sensitive to chemotherapy (EBCTCG, 2005), but are amenable to hormonal therapy. The most commonly used strategies for pharmacological inhibition of ER signalling are treatment with anti-oestrogens or aromatase inhibitors. For patients with metastatic disease, the response rate to first-line hormonal therapy with anti-oestrogens or aromatase inhibitors ranges from 21% to 33% (Nabholtz *et al*, 2000; Bonneterre *et al*, 2001; Mouridsen *et al*, 2001; Chia *et al*, 2008). The objective response rates of second-line hormonal therapies, such as exemestane or fulvestrant, measured by Response Evaluation Criteria in Solid Tumours (RECIST) criteria in a recent trial were 6.7% and 7.4%, respectively (Chia *et al*, 2008). A study of low dose estradiol as second- or third-line therapy showed stable disease, but no objective responses (Ellis *et al*, 2009). Thus, novel approaches to reverse hormone therapy resistance are needed.

Histone deacetylases (HDACs) and histone acetyltransferases have important roles in the maintenance and function of chromatin by regulating the acetylation of histones. Recent data suggest that HDACs and histone acetyltransferases regulate the acetylation of many non-histone targets and therefore may represent a key means of post-translational regulation beyond their established roles in transcriptional regulation. Biologically, HDAC inhibitors induce growth arrest, differentiation, and cell death in breast cancer cells. Despite clinical efficacy in patients with cutaneous T-cell lymphomas, the therapeutic window of the currently available HDAC inhibitors may not suffice for meaningful anti-tumour efficacy in breast cancer when used as a single agent without more careful patient selection or the definition of a biomarker (Luu *et al*, 2008).

In preclinical models, treatment of ER-positive breast cancer cells with HDAC inhibitors leads to transcriptional downregulation and protein modification of the ER (Yi *et al*, 2008). Treatment with an HDAC inhibitor reverses tamoxifen-induced ER stabilisation, which is followed by induction of pro-apoptotic genes and apoptotic cell death (Hodges-Gallagher *et al*, 2006; Bicaku *et al*, 2008; Thomas *et al*, 2011). Potentiation of tamoxifen by the HDAC inhibitor vorinostat has been shown in preclinical models at clinically achievable and tolerable concentrations (Kelly *et al*, 2005; Hodges-Gallagher *et al*, 2006; Galanis *et al*, 2009; Munster *et al*, 2009b). Epigenetic modulation of ER signalling by HDAC inhibitors may therefore represents a novel strategy to reverse hormone therapy resistance in advanced breast cancer. Further studies suggest that HDAC1 and 2 may have an important role in the regulation of oestrogen signalling and may therefore be relevant targets for HDAC inhibitor activity.

Prior studies with HDAC inhibitors suggest that these agents have a relatively short terminal half-life, with that of vorinostat reported to range from 21 to 58 min (Kelly *et al*, 2003). However, the pharmacodynamic effects often exceed the plasma half-life of these drugs, suggesting that pharmacodynamic measures may be a better predictor of tissue drug exposure than pharmacological values. Furthermore, several studies suggest vorinostat levels vary considerably between patients (Kelly *et al*, 2003; O’Connor *et al*, 2006). This may account for the poor correlation between vorinostat plasma levels and change in histone acetylation, a biomarker for molecular response (Munster *et al*, 2009b). A pharmacodynamic assay has therefore been developed by our laboratory to measure histone acetylation and HDAC enzyme expression in peripheral blood mononuclear cells (PBMCs) to allow for a more reliable means to measure the target activity of HDAC inhibitors in this study. Published data from a previous trial suggest that the change in histone acetylation in *in vitro* models, PBMCs, and tumour cells is comparable (Hodges-Gallagher *et al*, 2006; Bicaku *et al*, 2008).

Therefore, the objectives of this phase II trial were (1) to evaluate the toxicity of vorinostat and tamoxifen when administered in patients with hormone receptor-positive breast cancer after progression on prior hormone therapy, (2) to estimate the anti-tumour activity of vorinostat and tamoxifen in this patient population, and (3) to characterise the pharmacodynamic profile of histone acetylation and HDAC2 expression.

## Materials and methods

### Patient selection

Pre- and post-menopausal women with ER- or PgR-positive metastatic breast cancer with (1) progression on any number of aromatase inhibitors for metastatic disease or (2) recurrence of disease while on adjuvant aromatase inhibitors or (3) pre-menopausal women who had completed tamoxifen for at least 1 year were eligible for this trial who did not wish to undergo ovarian suppression in conjunction with aromatase inhibitor treatment. Prior treatment with tamoxifen or fulvestrant was permitted in the adjuvant setting, yet not for metastatic disease. Patients were allowed to have up to three prior chemotherapy regimens for metastatic disease. Bone-only disease was permissible if at least one lesion measured 1 cm by magnetic resonance imaging. Measurable disease, as defined by RECIST 1.0, was required. Other eligibility criteria were (1) Eastern Cooperative Group performance status of 0, 1, or 2; (2) adequate bone marrow function (white blood cell count ⩾3000 per mm^3^, absolute neutrophil count ⩾1500 per mm^3^, and platelets ⩾100 000 per mm^3^), adequate hepatic function (aspartate aminotransferase, alanine aminotransferase, and alkaline phosphatase levels ⩽2.5 times upper limit of institutional normal, total bilirubin ⩽2.0 mg dl^−1^), and adequate renal function (serum creatinine ⩽1.8 mg dl^−1^ or creatinine clearance >60 ml per minute times upper limit of institutional normal); (3) no prior treatment with HDAC inhibitors or other therapies for breast cancer within the preceding 3 weeks, (4) no prior radiation to the only measurable lesion; (5) no active intercurrent medical condition; (6) no vaginal bleeding, known endometrial hyperplasia, or cancer; and (7) no other invasive malignancies within the last 5 years, with the exception of non-melanoma skin cancer and *in situ* cervical cancer, (8) patients with brain metastases had to demonstrate stability for at least 90 days. Patients with prior thromboembolic events were to be on therapeutic anti-coagulation during the entire time of study.

All participants provided written informed consent, and participating institutions obtained annual Institutional Review Board approval in accordance with federal, state, local, and institutional requirements and guidelines.

### Drug administration, safety, and response assessment

Patients received 400 mg of vorinostat once daily for 21 of 28 days and 20 mg tamoxifen daily without interruption. Both drugs were given in an oral formulation. A cycle consisted of 28 days. Patients were allowed to remain on treatment until disease progression or the emergence of unacceptable toxicity. Toxicity assessment, interim history and physical exams, complete blood count, and serum chemistry profile chemistries (electrolytes, blood urea nitrogen, creatinine, magnesium, calcium, phosphate, aspartate aminotransferase, alkaline phosphatase, and total bilirubin) were obtained at baseline, weekly in cycle one, and then on day 1 of every cycle.

Tests for tumour markers and documentation of measurable disease by computed tomography (CT) to evaluate response were performed after every two cycles. Adverse events and other symptoms were graded according to the NCI Common Terminology Criteria of Adverse Events (CTCAE) version 3.0. Both agents were given at the full prescribing dose. As this was the first trial to combine vorinostat with tamoxifen, we performed additional safety evaluations during the enrolment of the first 15 patients with the mandate to halt the trial in the case where grade 3 and 4 toxicities were seen in 4 or more of the first 15 patients. A reduction to 75% of the vorinostat dose was required for patients experiencing grade 3 non-haematological toxicity and grade 4 haematological toxicity after recovery of toxicities with a second dose modification to 50%. If toxicities persisted, patients were withdrawn from the study. Consistent with standard practice, the tamoxifen dose was reduced for tamoxifen-specific toxicity only.

### Pharmacodynamics: histone acetylation and HDAC expression

Peripheral blood mononuclear cells obtained pre-treatment and day 8 post-treatment in cycle 1 were isolated by Ficoll centrifugation (Ficoll-Paque, GE HealthCare, Piscataway, NJ, USA). Cells were spun onto glass slides, fixed in 5% acetic acid/95% ethyl alcohol, and blocked with 2% bovine serum albumin. Histone H4 acetylation (rabbit polyclonal, Upstate Biotechnology, Lake Placid, NY, USA), and HDAC2 (monoclonal, Upstate Biotechnology) were visualised using fluorescently labelled secondary antibodies. Slides were counter stained with an antibody control directed against pan-histone H3 (monoclonal, BD Biosciences, Franklin Lakes, NJ, USA) for acetyl-H4 and polyclonal, Upstate Biotechnology for HDAC2) and evaluated by immunofluorescence microscopy (Zeiss Axio Imager.Z2, Carl Zeiss Inc., Thornwood, NY, USA). Resultant images were analysed as previously described (Munster *et al*, 2007, 2009a). Briefly, the mean pixel intensity for each epitope was determined within the nucleus from acquired images using Adobe Photoshop software. The mean pixel intensity for each epitope was determined adjacent to each cell and subtracted from the nuclear value to control for background staining. Pre- and post-treatment histone acetylation and HDAC2 expression were normalised to pan-histone H3 expression (Munster *et al*, 2007, 2009a). At least 100 cells were evaluated from two slides for each time point and condition. Changes in H4 histone acetylation were further verified by western blot when sufficient sample was available (Munster *et al*, 2007, 2009a). In brief, total cell extracts (10 *μ*g) were separated by SDS–PAGE and probed with primary antibodies against histone acetyl-H4 and pan-histone H3 (polyclonal, Upstate Biotechnology). Protein levels were quantified by densitometry using Image J software (NIH).

### Statistical design and methods

The study employed a Simon two-stage design with early stopping rules to preempt enrolment in the event of insufficient activity or excessive toxicity (Chen and Ng, 1998). During the first stage, 18 patients were to be enrolled and evaluated. If at least two responses were observed among the first 18 patients, an additional 25 patients were to be enrolled in a second phase. The regimen would be considered inactive if seven or fewer patients responded. If the ‘true’ response probability is 10%, the average probability to end the trial early would be 73.38%. Conversely, if the ‘true’ response probability is 25%, there is a 13.53% probability to incorrectly classify the trial as inactive. Pearson's correlation coefficient methods were used to estimate correlations between two variables and to perform the test of significance of the estimated correlation with two-sided *P*-values at a 0.05 significance level (SAS version 9.1, SAS Institute Inc., Cary, NC, USA). Early stopping rules were set for dose-limiting toxicities to occur in no more than 33% of patients in the first cycle.

## Results

### Patient characteristics

The study (clinicaltrials.gov identifier number NCT00365599) enrolled 43 evaluable patients at the Moffitt Cancer Center in Tampa, Florida and the University of California, San Francisco. One patient withdrew consent after 2 weeks and had to be replaced as pre-specified by the protocol. Patient characteristics and demographics are listed in Table 1.

### Adverse events

A summary of adverse events by CTCAE (version 3.0) for all the patients is listed in Table 2. As in standard medical practice, dose reduction for tamoxifen was not performed for any toxicities, but tamoxifen was stopped in one patient after she developed a thromboembolic event. Vorinostat was given 21 of 28 days of each cycle, whereas tamoxifen was given continuously. This schedule allowed for the distinction between vorinostat toxicities and toxicities associated with the combination. The predominant toxicities of vorinostat included fatigue and anorexia, neutropenia, lymphopenia, and thrombocytopenia. Dose adjustments were made for all patients with drug-related grade 3 and grade 4 toxicities associated with vorinostat. Dose adjustments to 300 mg vorinostat for grade 3 toxicities in 13 patients (30%) and to 200 mg for grade 4 toxicities or persistent grade 3 toxicities in 7 patients (16%) were required. These included four (9%) patients requiring dose reductions for neutropenia as the initial event, four (9%) for thrombocytopenia, seven (16%) for fatigue, and one each (2%) for weight loss, liver enzyme elevation, and diarrhoea. Three patients experienced pulmonary emboli, two of which were incidental findings on CT scans. One patient had a symptomatic pulmonary embolus after a long transcontinental flight. In 13 of the 20 patients, dose reductions were required after the first cycle. Alopecia occurred in four patients after prolonged exposure (cycle 4+). Alopecia is rarely seen with tamoxifen alone, but has been reported in occasional cases with vorinostat. While a significant number of patients had to be dose adjusted, another subset tolerated long-term administration of the drug combination for 2 years or longer.

### Anti-tumour efficacy of vorinostat and tamoxifen

Responses were assessed by RECIST criteria version 1.0 (Therasse *et al*, 2000). Patients with bone-only disease were required to have at least one lesion that measured 1 cm and was measurable by CT or magnetic resonance imaging. The study proceeded through the second stage after confirmed objective responses were seen in 4 of the first 18 patients, and grade 3 and 4 toxicities in <4 patients. Confirmed objective responses by RECIST criteria were seen in 8 out of 43 (19%) patients and stable disease for ⩾24 weeks in 9 out of 43 (21%) patients. Two of the patients with stable disease showed a complete metabolic response with disappearance of FGD-PET avidity after an initially increased metabolic activity by fluoro-deoxy glucose (FDG) positron emission tomogram (PET). The median number of cycles delivered was four. The time to progression was 10.3 months (6–30+ months) with an overall median survival of 29 months (95% confidence interval (95% CI): 20–38.5 months). At the time of this report, two patients remain on study treatment.

All of the patients with a response or clinical benefit had progressed on at least one prior aromatase inhibitor, and 8 out of 17 (47%) patients had received prior adjuvant tamoxifen. Responses were seen in patients with or without visceral disease, and responses were maintained in patients despite the need for dose adjustments in 8 out of 17 patients. Hormone receptor expression and prior treatment history of the responders are depicted in Table 3.

### Pharmacodynamics

**Figure 1:**
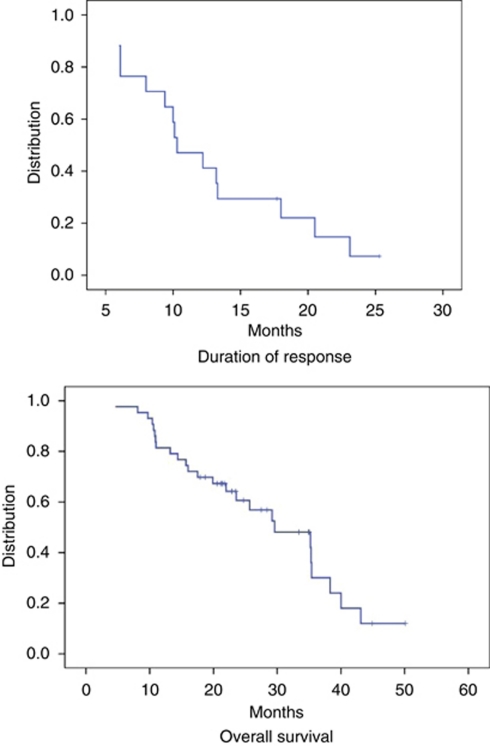
Median duration of response and overall survival.

**Figure 2:**
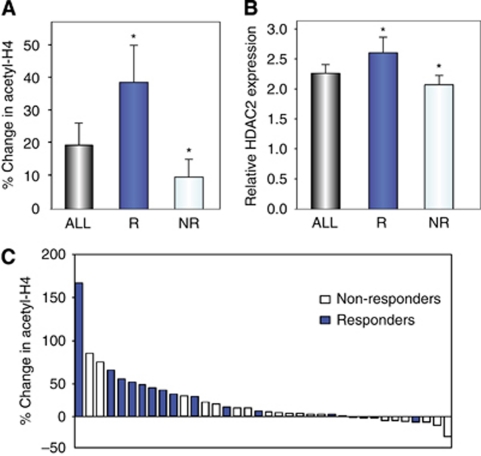
Percent change in acetyl-H4 (day 8 post-treatment pre-dose) in PBMCs by immunofluorescence normalised to pan-histone H3 expression. (**A**) Mean change of acetyl-H4 and (**B)** mean baseline HDAC2 expression relative to pan-histone H3 expression in all patients, in responders (R; response or clinical benefit ⩾24 weeks) and in non-responders (NR). ^*^Statistical significance for acetyl-H4, *P*=0.022 (R *vs* NR) and HDAC2, *P*=0.04 (R *vs* NR). (**C**) Waterfall plot of acetyl-H4 in (▪) responders and (□) non-responders (*n*=36).

Previous studies with vorinostat and valproic acid in patients with solid tumour malignancies suggested that the induction of histone H4 acetylation in tumour cells was comparable to the increase in histone H4 acetylation seen in PBMCs (Munster *et al*, 2007, 2009a). Similarly, the drug-induced changes in histone acetylation in tumour and PBMCs mimicked those seen in cultured breast cancer cells treated with vorinostat at comparable doses. Prior studies further suggested that the assessment of histone acetylation required much less tissue or fewer cells when using immunofluorescence and was feasible in tumour tissues obtained by fine needle administration. Prior studies suggested that histone H4 acetylation was more robust than histone H3 acetylation (Munster *et al*, 2007, 2009a). Hence for this study, changes in histone H4 acetylation in PBMCs were measured by immunofluorescence first and repeated by western blot if sufficient material was available. Complete pre- and day 8 post-treatment samples were available in 36 out of 43 patients (Figures 1 and 2). Missing data sets were in part due to the insufficient number of PBMCs as a result of myelosuppression in a subset of the patients, which happened early in the course of treatment. The mean increase in histone H4 acetylation was 20% over baseline and normalised for pan-histone H3 expression (CI: 14–26%) for the entire group. In patients with a partial response or stable disease for >24 weeks, the treatment-induced mean change in H4 histone acetylation was 39% (CI: 26–51%) compared with 10%. In our experience, the changes in histone acetylation by immunofluorescence were typically less pronounced than those seen by western blot analysis. To confirm the correlation of increased histone acetylation in responding *vs* non-responding patients, pre- and post-treatment samples were assessed for histone acetylation changes by western blot analysis. Consistent with the immunofluorescence analysis, statistically more pronounced histone H4 acetylation was found in patients with a response or stable disease (430% increase (CI: 219–633)) *vs* non-responders (6% (CI: −7–21), *P*=0.042). As seen with our prior studies, the numerical changes were more robust, the number of patients with no change in histone acetylation were almost identical. However, immunofluorescence requires much less tissue and allows the assessment of histone acetylation in tumours cells obtained by fine needle aspiration (data not shown).

While several reports suggest a number of HDAC inhibitor targets, both histone and non-histone, as relevant for their observed anti-tumour activity, our preclinical data suggest that the depletion of HDAC2 by siRNA is sufficient, as its depletion mimics the effects of an HDAC inhibitor–tamoxifen combination *in vitro*. Thus, we believe HDAC2 represents the relevant target for achieving synergy with tamoxifen. We have further shown that the select depletion of HDAC2 may be important in the modification of ER signalling (Bicaku *et al*, 2008). Similarly, baseline expression of HDAC2 was higher in responding *vs* non-responding patients. Measured in relative expression to pan-histone H3 expression, baseline HDAC2 expression in responders (R) was 2.48 times compared with 1.88 times in non-responders (NR) (*P*=0.040, Figure 2B).

**Figure 3:**
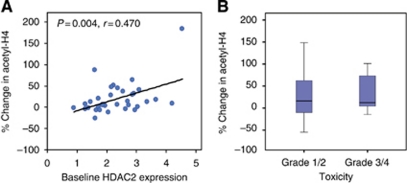
(**A**) Scatter plot of baseline histone deacetylase 2 (HDAC2) expression *vs* percent change in acetyl-H4 in PBMCs (day 8 post-treatment pre-dose) by immunofluorescence normalised to pan-histone H3 expression, analysed by Pearson's correlation (*P*=0.004, correlation coefficient of 0.470). (**B**) Percent change in acetyl-H4 in patients with less severe (grade 1/2) and more severe (grade 3/4) haematological or non-haematological toxicities (*P*=0.49).

In previous studies, we have shown that the change in histone acetylation correlates with baseline expression of HDAC2 (Munster *et al*, 2007, 2009a) in PBMC and tumour cells. In this study, higher baseline expression of HDAC2 in PBMCs was associated with a more pronounced increase in histone H4 acetylation (Figure 3A) (*P*=0.003, Pearson's correlation coefficient=0.519).

Dose adjustments were required in 20 (47%) of 43 treated patients. To determine whether the change in acetylation was predictive of toxicity, acetylation in patients with grade 3 and 4 toxicities (*n*=20) was plotted against acetylation in those with grade 1 and 2 toxicities (*n*=23) only. Changes in acetyl-H4 were not associated with severity of haematological or non-haematological toxicity (*P*=0.49, Figure 3B).

## Discussion

This is the first clinical study to evaluate the benefits of combining an HDAC inhibitor with an anti-oestrogen in patients with advanced ER-positive breast cancer. The rationale for this combination stems from extensive preclinical data suggesting epigenetic modulation and post-translational modification of the ER by HDAC inhibitors enhances the anti-tumour effects of tamoxifen (Yang *et al*, 2001; Kawai *et al*, 2003; Alao *et al*, 2004; Jang *et al*, 2004; Saji *et al*, 2005; Kawai and Arinze, 2006; Sharma *et al*, 2006; Fiskus *et al*, 2007; Zhou *et al*, 2007; Bicaku *et al*, 2008). We found that treatment of cultured breast cancer cells with vorinostat leads to downregulation and reversal of tamoxifen-induced stabilisation of the ER (Bicaku *et al*, 2008). The anti-tumour activity of tamoxifen is primarily anti-proliferative. In the presence of an HDAC inhibitor, however, we find that tamoxifen induces apoptosis rather than growth arrest. Further studies suggest that this interaction is mediated through inhibition of HDAC2 (Munster *et al*, 2007, 2009b; Bicaku *et al*, 2008; Marchion *et al*, 2009). Select depletion of HDAC2 by small interfering RNAs mimics the effects of an HDAC inhibitor on the ER and its downstream signalling (Bicaku *et al*, 2008). This led us to pursue a clinical trial evaluating the addition of an HDAC inhibitor to tamoxifen for the treatment of women with ER-positive breast cancer who had progressed on prior hormonal therapy.

Vorinostat was the first HDAC inhibitor approved by the FDA for the treatment of cancer, specifically cutaneous T-cell lymphoma. In addition to the effects in patients with cutaneous T-cell lymphoma, HDAC inhibitors appear to be active in Hodgkin's lymphoma and other haematological malignancies (Duvic *et al*, 2007). In contrast, the anti-tumour effects of vorinostat in solid tumour malignancies have been less evident. Vorinostat has been tested as a single agent in patients with metastatic breast cancer. Although disease stabilisation was observed in 30% of the patients, no clinical responses were achieved (Luu *et al*, 2008). Similarly, despite reported efficacy as single agents in several preclinical models, clinical benefit with HDAC inhibitors in several other solid tumour malignancies has been modest, limited mostly to disease stabilisation. Preclinical data from our laboratory and others suggest that HDAC inhibitors have the ability to re-sensitise tamoxifen-resistant cells to hormone therapy, and has been hypothesised to prevent the emergence of hormone therapy resistance (Yang *et al*, 2001; Zhou *et al*, 2007; Thomas *et al*, 2011).

This study's findings suggest that the addition of the HDAC inhibitor vorinostat to tamoxifen results in durable responses (8 out of 43, 19%) and prolonged disease stabilisation (9 out of 43, 21%) in patients who had progressed on at least one prior aromatase inhibitor. Further, more than half of the patients had previously progressed on adjuvant tamoxifen. A significant proportion of the patients also received chemotherapy. The expected response rate in this patient population, based on the EFECT trial, is 7.6% for fulvestrant and 6.7% for exemestane (Chia *et al*, 2008). Two more contemporary trials suggested that while stable disease is achievable, objective, and durable responses are rare. The first trial reported disease stabilisation, but no objective responses using estradiol in this setting (Ellis *et al*, 2009). The second trial comparing tamoxifen plus placebo *vs* tamoxifen plus EGFR inhibitor gefitinib demonstrated that tamoxifen and placebo treatment resulted in a 15% objective response rate in patients with newly diagnosed metastatic breast cancer or recurrent disease after adjuvant tamoxifen. However, in the stratum best comparable to the patient population in this study, patients recurring on or not responding to prior aromatase therapy, no objective responses were observed (Osborne *et al*, 2011). While in this study, the observed anti-tumour activity of 19% confirmed partial responses by RECIST criteria in this heavily pre-treated patient population is therefore very encouraging. Table 3 shows that responses were seen in patients after progression on two or three prior endocrine therapies and chemotherapy.

Furthermore, the correlative studies accompanying this clinical trial suggest that enrichment of responsive patients may be feasible. The pharmacological effects of vorinostat were evaluated in PBMCs. A statistically significant increase in histone H4 acetylation compared with baseline was observed in only 21 out of 36 (58%) patients (Figure 2). This suggests that 15 out of 36 patients (42% of the evaluated patients) did not reach vorinostat plasma levels that were high enough to induce a change in histone acetylation, or did not express the appropriate histone target. Several studies with vorinostat have shown that histone acetylation occurs at lower concentrations than those required for the modulation of other targets. Our data suggest that day 8 histone acetylation is a strong predictor of response. We have shown previously that vorinostat-induced histone acetylation in PBMCs is comparable to histone acetylation in tumour cells (Munster *et al*, 2009a). The mean increase in histone H4 acetylation for all patients in this study was 20% (95% CI: 14–26%) over baseline when treated with 400 mg of vorinostat. This may appear lower than has been previously reported when histone acetylation was measured by western blot analysis (Kelly *et al*, 2003). The effects, however, were comparable to our findings from other studies where histone acetylation was measured by immunofluorescence. We found that the observed changes in histone acetylation induced by 400 mg oral vorinostat given daily in this study were comparable to the results observed in a dose escalation phase I trial evaluating vorinostat in combination with the anthracycline doxorubicin, conducted by our group (Munster *et al*, 2009a). We reported a 25% increase in mean histone acetylation (95% CI: 3–47) on day 3 in patients receiving 400 mg of vorinostat, with mean plasma levels of 114 nM (95% CI: 37–191). Patients treated with higher doses of the HDAC inhibitor resulted in a 20% increase in median H4 histone acetylation (95% CI: 29–70) at 600 mg of vorinostat, and 84% (95% CI: 54–114) for 800 mg. Corresponding mean vorinostat levels were 256 nM (95% CI: 133–378) and 760 nM (95% CI: 598–924), respectively (Munster *et al*, 2009a).

To confirm that certain patients did not show a change in acetylation, we measured histone modification changes alternatively by western blot analysis. Although the range of histone acetylation measured by western blot analysis was numerically greater, a significant change in acetyl-H4 was only observed in 57% of the patients, comparable to the findings of the immunofluorescence analysis.

Furthermore, as shown in previous studies, baseline expression of HDAC2 is positively correlated with a change in histone H4 acetylation (Figure 3A), suggesting HDAC2 is a potential biomarker and important pharmacological target of vorinostat.

Responders were also more likely to exhibit elevated histone acetylation following treatment and increased baseline HDAC2 expression (Table 3; Figures 2A and B). An increase in histone acetylation was observed in 13 of 14 evaluable responders (Figure 2C). Thus, by identifying hyperacetylators at the initiation of treatment, patients most likely to benefit from this treatment would be enriched. Furthermore, determining initial acetylation response may provide direction for vorinostat dose modification.

Vorinostat and tamoxifen treatment was well tolerated by many of the patients and long-term exposure of up to 2 years was feasible. A subgroup of patients required dose modifications, however, due to grade 3 and 4 toxicities. In addition to myelosuppression, the predominant toxicities were fatigue and anorexia. These toxicities have been well described for vorinostat, and are less likely due to an interaction between vorinostat and tamoxifen. In the fourth week of each cycle (tamoxifen alone treatment), patients reported temporary alleviation from nausea, fatigue, anorexia, and myelosuppression. Notably, dose modification did not appear to compromise response, as 8 of the 17 patients with clinical benefit were dose adjusted, and maintained their response. Observed toxicities did not correlate with changes in histone acetylation (Figure 3), suggesting they may be the result of off target effects.

In support of our preclinical findings, baseline HDAC2 expression in patients’ PBMCs correlated with the degree of histone acetylation. Thus, the assessment of baseline HDAC2 expression may predict a patient's molecular response. Patients with low HDAC2 expression, or those who do not show a change in acetylation, could then be removed from study or receive higher doses of the HDAC inhibitor if feasible.

In summary, this trial suggests that the addition of vorinostat to tamoxifen in patients with hormone receptor-positive breast cancer results in tumour regression or prolonged disease stabilisation in 40% of the patients who had progressed on prior hormonal therapy and chemotherapy. Although the current study may be limited by its sample size, the results are greater than those of contemporary studies testing endocrine therapy in second- and third-line therapy with response rates reported in <10% of the patients, or no responses when measured by RECIST criteria (Chia *et al*, 2008). However, a randomised trial is required to determine the effects of the combination over the potential efficacy of tamoxifen alone. Pharmacodynamic assessment of vorinostat-induced histone H4 acetylation and HDAC2 expression at baseline was strong predictors of biological activity and clinical benefit. This suggests that the absence of histone acetylation could be used as an early negative predictor for patients who are not likely to benefit. These patients could then be removed from study, or be treated with a higher dose. The observed anti-tumour efficacy warrants further testing of HDAC2 inhibitors and hormonal therapy, yet the development of rapid acetylation bioassays and assessment of baseline HDAC2 expression in tumours in future studies may provide a feasible method to enrich for patients more likely to benefit.

## Figures and Tables

**Table 1:** Patient demographics, tumour characteristics, and treatment history

**Characteristic**	
*Number of patients*	43
Measurable disease, *n* (%)	43 (100%)
	
*Age, median (range), years*	56 (33–71)
<65	37 (86%)
>65	6 (14%)
	
*Race and ethnicity, n (%)*	
Caucasian	36 (84%)
Asian	5 (12%)
African American	2 (5%)
Non Hispanic	39 (91%)
Hispanic	4 (9%)
Performance status, median (range)	1 (0–2)
Body mass index, kg m^−2^, median (range)	27.9 (20.7–44.2)
	
*Visceral disease, n (%)*	
Yes	32 (74%)
No	11 (26%)
	
*Hormone receptor status, n (%)*	
ER+/PR+	25 (58%)
ER+/PR−	17 (40%)
ER+/PR unknown	1 (2%)
ER−/PR+	0 (0%)
	
*HER amplification, n (%)*	
Amplified	5 (12%)
Non-amplified	38 (88%)
	
*Prior endocrine therapy for metastatic disease, n (%)*	
0 prior endocrine	3 (7%)
1 prior endocrine therapy	25 (58%)
2 prior endocrine therapies	15 (35%)
	
*Adjuvant endocrine therapy, n (%)*	34 (79%)
Tamoxifen	25 (58%)
Aromatase inhibitor	16 (37%)
Aromatase inhibitor for metastatic disease	35 (81%)
	
*Prior chemotherapy for metastatic disease, n (%)*	28 (65%)
Regimens: median (range)	1 (0–3)
Cycles delivered, median (range)	4 (1–27+)

Abbreviations: ER=oestrogen receptor; PR=progesterone receptor; HER=human epidermal growth factor receptor.

**Table 2:** Common Terminology Criteria of Adverse Events, grades (2–4), by number of patients (%)

	**Grade 2**	**Grade 3**	**Grade 4**
*Non-haematological toxicities (*N*=43)*			
Fatigue	8 (19%)	7 (16%)	
Nausea	8 (19%)	2 (5%)	
Vomiting	2 (5%)	2 (5%)	
Diarrhoea	7 (16%)	1 (2%)	
DVT/PE			3 (7%)
Anorexia/weight loss	7 (16%)	4 (9%)	—
Mucositis	2 (5%)	1 (2%)	—
Hepatic dysfunction (ALT/AST)	3 (7%)	1 (2%)	—
Hyperglycaemia	5 (12%)	1 (2%)	—
Alopecia	4 (9%)	—	—
			
*Haematological toxicities (*N*=43)*			
Thrombocytopenia	4 (9%)	3 (7%)	1 (2%)
Leukopenia	4 (9%)	1 (2%)	—
Neutropenia	5 (12%)	7 (16%)	—
Lymphopenia	5 (12%)	6 (14%)	—
Anaemia	2 (5%)	—	—

Abbreviations: ALT=alanine aminotransferase; AST=aspartate aminotransferase; DVT=deep-vein thrombosis; PE=pulmonary embolism.

**Table 3:** Tumour characteristics, prior treatment history, and dose modifications in patients with a response or clinical benefit

	**Hormone receptor status**	**HER2 status**	**Visceral disease**	**Prior aromatase inhibitors**	**Prior adjuvant tamoxifen**	**AC-H4**	**Dose modification, mg**
*Patients with partial response*							
Patient 1	ER+/PR+	Not ampl	Yes	Letrozole	No	Yes	
Patient 2	ER+/PR+	Not ampl	Yes	Anastrozole, exemestane	Yes	Yes	300
Patient 3	ER+/PR+	Not ampl	Yes	Anastrozole, exemestane	Yes	No	200
Patient 4	ER+/PR−	Not ampl	Yes	Letrozole, exemestane	No	Yes	300
Patient 5	ER+/PR+	Not ampl	No	Anastrozole	No	Yes	300
Patient 6	ER+/PR+	Not ampl	Yes	Letrozole, exemestane	Yes	Yes	
Patient 7	ER+/PR+	Not ampl	Yes	Anastrozole, exemestane	No	ND	
Patient 8	ER+/PR+	Not ampl	No	Letrozole	No	Yes	
							
*Patients with stable disease ⩾ 24 weeks*							
Patient 1	ER+/PR−	Not ampl	No	Letrozole, anastrozole	Yes	Yes	
Patient 2	ER+/PR+	Not ampl	Yes	Letrozole, exemestane,	Yes	Yes	200
Patient 3	ER+/PR−	Not ampl	Yes	Anastrozole, letrozole	No	ND	
Patient 4	ER+/PR+	Not ampl	No	Letrozole	Yes	Yes	200
Patient 5	ER+/PR−	Not ampl	Yes	Anastrozole	No	Yes	
Patient 6	ER+/PR−	Not ampl	Yes	Anastrozole, letrozole	No	ND	
Patient 7	ER+/PR+	Ampl	No	Letrozole, exemestane	No	Yes	300
Patient 8	ER+/PR+	Not ampl	Yes	Letrozole	Yes	Yes	
Patient 9	ER+/PR+	Not ampl	No	Letrozole	Yes	Yes	300

Abbreviations: Ampl=amplified; HER2=human epidermal growth factor receptor 2; AC-H4=change in acetyl-H4 expression; ND=no data; ER=oestrogen receptor; PR=progesterone receptor.
